# Dr. Turner Anderson

**Published:** 1908-11

**Authors:** 


Dr. Turner Anderson, of Louisville, one of the prominent physicians in Kentucky, died at Saints Mary and Elizabeth Hospital in that city, October 13, 1908, aged 66 years. His disease is alleged to have been an affection of the heart.

